# The Biocontrol Efficacy of* Streptomyces pratensis* LMM15 on* Botrytis cinerea* in Tomato

**DOI:** 10.1155/2017/9486794

**Published:** 2017-11-28

**Authors:** Qinggui Lian, Jing Zhang, Liang Gan, Qing Ma, Zhaofeng Zong, Yang Wang

**Affiliations:** College of Plant Protection, Northwest A&F University, Yangling, Shaanxi 712100, China

## Abstract

LMM15, an actinomycete with broad spectrum antifungal activity, was isolated from a diseased tomato leaf using the baiting technique. A phylogenetic tree analysis based on similarity percentage of 16S rDNA sequences showed that the bacterium was 97.0% affiliated with the species* Streptomyces pratensis*. This strain was therefore coded as* S. pratensis* LMM15. The ferment filtrate of LMM15 had ability to inhibit mycelia growth of* Botrytis cinerea* and reduce lesion expansion of gray mold on detached leaves and fruits. In greenhouse experiments, both the fresh and dry weights of tomato seedlings were significantly increased with the increased concentrations of total chlorophyll. The incidence of tomato gray mold decreased by 46.35%; this was associated with the increase of proline content and malondialdehyde (MDA) and the changes in defense-related enzymes on tomato leaves when the strain was sprayed on the tomato leaves 24 h prior to inoculation with pathogens. This study showed that the strain* S. pratensis* LMM15 could be a potential agent for controlling tomato gray mold.

## 1. Introduction

Tomato gray mold, caused by* Botrytis cinerea* pers. ex., is one of the most destructive diseases to tomato (*Lycopersicon esculentum* L.) and is found in almost all greenhouses [1, 2]. This pathogen has the ability to infect tomato fruits, seeds, and plant bodies including flowers, leaves, and stems [3, 4]. The most important environmental factors normally are high relative humidity, moisture, and favorable temperature, all of which promote the infection by* B. cinerea* [5].

The primary strategy to control tomato gray mold is the use of fungicides [3, 4]. Many families of fungicides have been applied to manage tomato gray mold [6], such as benzimidazoles (e.g., carbendazim, thiophanate-methyl), dicarboximides (e.g., iprodione, procymidone, and vinclozolin), anilinopyrimidines (e.g., cyprodinil, mepanipyrim, and pyrimethanil), and N-phenylcarbamates (e.g., diethofencarb). However,* B. cinerea* has a great ability to produce resistance to fungicide, causing the intensive use of fungicide in greenhouses [7–13]. This may be an extremely important limiting factor in chemical control [1, 2]. In addition, more consumers are demanding food safety today. So more environment-friendly measures have to be found to control tomato gray mold.

Molecular engineering of tomato plants with the stilbene synthase, the key enzyme for the biosynthesis of the phytoalexin resveratrol, could represent an interesting alternative to the use of fungicides for controlling gray mold [14]. On the other hand, some natural products have undergone research, such as (poly-D-glucosamine) and plant-origin oligosaccharides; they have the ability to elicit defense responses and/or induce protection against* B. cinerea* in tomatoes and other crops or vegetables [15–18]. Meanwhile, Abro et al. [19] reported that nitrogen (N) fertilization had the ability to strengthen the control efficacy of the biocontrol agents (BCAs)* Trichoderma atroviride* and* Microdochium dimerum*. Although N fertilization was a promising method to control tomato gray mold, the key was BCAs.

Biological control of* B. cinerea* has been broadly researched [19–28]. Research has shown that the marine yeast* Rhodosporidium paludigenum* has a significant control efficacy in postharvest tomato fruits via nutrient competition. Wang et al. [29] and Dik and Elad [30] discovered that, in some cases, the biocontrol agents were more effective than the broad-spectrum fungicide tolylfluanid and the selective fungicide iprodione. Climatic conditions did not strongly influence the efficacy of the biocontrol agents, but regression analysis showed that high temperature during the day and a high vapor pressure deficit during the night reduced biocontrol efficacy [30].

Currently, there are many studies about BCAs preventing gray mold, but few studies have been carried out investigating the influence of BCAs on tomato defense systems under* B. cinerea*. The objectives of this study are to explore the biocontrol efficacy of the* Streptomyces* strain LMM15 against gray mold, determine the plant-growth-promoting effects, examine any impacts on the physiological functions of the tomato plants, and explain the impact on the tomato defense system of LMM15 when released from the stress of gray mold.

## 2. Materials and Methods

### 2.1. *Streptomyces*, Fungi, and Plant Materials

The strain LMM15 was isolated from naturally infected leaves of tomatoes in a greenhouse at Weinan, Shaanxi Province. The* B. cinerea* strain B05 was provided by the laboratory of integrated control of plant diseases in Northwest A&F University, China. Both strains were cryopreserved by freezing the suspension of conidia in 25% (vol/vol) glycerol at −80°C. Before use, strain LMM15 was transferred to Gause's No. 1 agar at 27°C for 7 days, while the* B. cinerea* was transferred to potato dextrose agar (PDA) at 25°C for 5 days. The LMM15 colony was transferred into Gause's No. 1 broth with a sterile needle (Φ = 5 mm) and incubated at 27°C for 120 h with shaking in the orbital incubator at 150 rpm·min^−1^. The concentration of LMM15 broth was then counted with a haemocytometer and adjusted to 10^7^ colony forming units (CFU) per milliliter [11]. The* Streptomyces* broth was filtered through 0.2 *μ*m Acrodisc Syringe Filters (Pall Corporation) to get a cell-free filtrate solution.

The* B. cinerea* cultures were prepared in PDA medium, incubated at 25°C for 7 days. And the conidia of* B. cinerea* were produced by a modification method which was previously described [31, 32]. After a 10-day incubation on the PDA plates, the conidia were rinsed from the surface of the plate with sterile distilled water; the concentration of the suspension was then adjusted to approximately 10^6^ conidia·mL^−1^. This suspension was supplemented with 10% glycerol and placed in a −20°C freezer for storage.

Seeds of the tomato cultivar “Maofen-8” were selected and grown in a nonsterilized mixture of soil and organic soil (1 : 1, v/v) in a greenhouse at 25°C ± 3°C and relative humidity of 75% with a 12 h light and dark cycle.

### 2.2. Effects of* Streptomyces* LMM15 on Mycelial Growth of* B. cinerea*

The effects of* Streptomyces* LMM15 on the mycelial growth of* B. cinerea* were assayed in PDA and potato dextrose broth (PDB). PDA was autoclaved and cooled naturally to approximately 25°C. The cell-free filtrate solution (CFFS) of strain LMM15 was mixed with the sterile to-be-solidified PDA to a final concentration of 10%. The PDA with the cell-free filtrate solution (CFFS) was then poured into Petri plates before being inoculated with 5 mm plugs from 6-day-old cultures of* B. cinerea* in the center of the plate. Sterile distilled water was used as the control where appropriate. The plates were incubated at 25°C [33], and the colony diameters were measured every 12 h after inoculation.

In addition, the cell-free filtrate solution (CFFS) of strain LMM15 was mixed with the sterile PDB to obtain final concentrations of 5%, 2%, and 1%. A 100 mL aliquot of PDB was poured into a 250 mL Erlenmeyer flask; then 5 mm plugs of* B. cinerea* were inoculated into the flask and incubated at 25°C for 3 d with constant shaking at 150 rpm. Sterile distilled water was used as the control where appropriate. The mixture was filtered by a Buchner funnel to obtain the mycelium before being dried at 65°C. The mycelium dry weights were weighed and the inhibition rates were calculated. Each treatment was repeated three times, respectively.

### 2.3. The Biocontrol Efficacy In Vivo

Tomato fruits of the cultivar “Maofen-8” were harvested from the greenhouse in the west of Yangling, Shaanxi, early in the morning and quickly taken to the laboratory. The fruits were selected on the basis of color, size, and absence of physical injury. They were then randomly grouped into clusters of nine fruits. The tomato fruit samples were wounded (approximately 5 mm deep) at the equator using a sterile syringe needle (three sites/fruit; six fruits/treatment). Then the wounds were then treated with (1) pretreatment: 10 *μ*L of cell suspensions of LMM15 (1 × 10^6^ cells/mL) in advance and 24 h later 10 *μ*L of* B. cinerea* conidia suspension (1 × 10^6^ conidia/mL) were inoculated into each vulnus, respectively; (2) cotreatment: 10 *μ*L of cell suspensions of LMM15 (1 × 10^6^ cells/mL) and 10 *μ*L of* B. cinerea* conidia suspension (1 × 10^6^ conidia/mL) were inoculated into each vulnus simultaneously; and (3) postpone-treatment: 10 *μ*L of* B. cinerea* conidia suspension (1 × 10^6^ conidia/mL) in advance and 24 h later 10 *μ*L of cell suspensions of LMM15 (1 × 10^6^ cells/mL) were inoculated into each vulnus, respectively. 10 *μ*L of sterile distilled water or procymidone was used as the control. The procymidone was applied at the concentration suggested by the manufacturers (approximately diluted 1000 times). Each treatment was repeated three times.

Leaves picked from 10-week-old greenhouse-grown tomato (cv. Maofen-8) plants were laid in sterile Petri dishes containing moistened gauze and each leaf was inoculated with three plugs of* B. cinerea* (*ϕ* = 5-mm mycelia) 24 h before (pretreatment) or after (postpone-treatment) or simultaneously (cotreatment) inoculated with 10 *μ*L of cell suspensions of LMM15 (1 × 10^6^ cells/mL) at each site. Sterile distilled water or procymidone was used as the control where appropriate. The procymidone was applied at the concentration suggested by the manufacturers (approximately diluted 1000 times). Each treatment was repeated three times.

All the samples were stored in single enclosed plastic bags, respectively, and incubated at 22°C for 3 days; the relative humidity was maintained at over 95%. The relative lesion diameters were measured with a slide caliper rule and the lesion area was calculated. The biocontrol efficacy of LMM15 was calculated as follows:

(1) $$\begin{array}{c} \text{Control efficacy}=\sum \frac{\left(\text{the mean lesion area of the control}-\text{the mean lesion area of the treatment}\right)}{\text{the mean lesion area of the control}}\times 100\%. \end{array}$$

### 2.4. Effects of* Streptomyces* LMM15 on Improvement of Plants Growth

Tomato (cv. Maofem-8) was grown as previously described. When the seedlings were at trefoil stage, the fermentation broth of LMM15 and water (as control) was root-irrigated with an amount of 10 mL per individual plant. Inoculated tomato plants were maintained in a greenhouse at 22°C. The plant height, stem diameter, the ratio of dry weight/fresh weight (DW/FW) [34], and the total chlorophyll content were measured as previously described [35] 45 d after inoculation. For the treatment, three replicates were used.

### 2.5. The Biocontrol Efficacy of LMM15 on Tomato Gray Mold

To determine the biocontrol efficacy of strain LMM15, tomato (cv. Maofem-8) seedlings (3 true leaf) were transplanted at the trefoil stage in plastic pots (10 cm × 10 cm × 15 cm) containing a soil mixture. Then the fermentation broth of LMM15, procymidone (negative control, at the concentration suggested by the manufacturers), and water (as control) was sprayed until there was runoff on the tomato seedlings. Each treatment consisted of nine plants and the two treatments had three replications. The spray timing treatments were performed as described above: (1) pretreatment, (2) cotreatment, and (3) postpone-treatment. For the fungal inoculation, the conidia suspension of* B. cinerea* was sprayed until there was runoff. Tomato seedlings were maintained in a greenhouse at 25°C ± 3°C and relative humidity of 90% with a 12 h light and dark cycle. Each treatment consisted of nine plants. Gray mold symptoms were recorded 30 d after inoculation and the disease incidence of each treatment was calculated as the percentage of diseased plants, while the disease severity of each treatment was assessed by the percent of leaves that showed gray mold symptoms to total number of leaves of each plant according to the following scales: 0, no diseased leaves; 1, ≤5%; 3, 6%–10%; 5, 11%–25%; 7, 26%–50%; 9, >50% diseased leaves. Disease index and the biocontrol efficacy of LMM15 were calculated as follows:

(2) Disease index=∑disease severity score×number of leaves with the scalethe highest severity score×the total number of leaves examined×100Control efficacy=∑the mean disease index of the control−the mean disease index of the treatmentthe mean disease index of the control×100%.

### 2.6. The Biocontrol Analysis on Tomato Plant

The activities of defense-related enzymes and proline content or the lipid peroxidation (MDA content) as well as plant height, stem diameter, the ratio of DW/FW, and the total chlorophyll content of plants were subsequently measured. Operational sequences were accomplished at 4°C. Five grams of leaf samples was grounded mixed with 25 mL of precooling sodium phosphate buffer (50 mmol·L^−1^, 4°C, pH 7.8) containing 1% polyvinylpyrrolidone (PVP) and 1.33 mmol/L EDTA-Na for superoxide dismutase (SOD), peroxidase (POD), phenylalanine ammonia lyase (PAL), and proline content as well as the lipid peroxidation (MDA content), respectively. The homogenates were then centrifuged at 12,000*g* for 15 min at 4°C and the supernatants were assayed as previously described [36–40]. The experiment was repeated three times.

### 2.7. Identification by 16S rDNA Gene Sequence Analysis

For analysis of the 16S rDNA sequence, the DNA of strain LMM15 was isolated by the method previously described [41]. The universal primers used in this research were (27f) 5′-AGAGTTTGATCCTGGCTC-3′ [42] and (1492r) 5′-CGGCTACCTTGTTA- CGACTT-3′ [43]. The PCR reaction mixture system contained 1 *μ*L of template DNA, 2 *μ*L of reverse primers and 2 *μ*L of forward primers (30 pmol *μ*L^−1^), 4 *μ*L of dNTP mix, 5 *μ*L of 10x PCR buffer containing 1 *μ*L of Taq DNA polymerase (5 U *μ*L^−1^), 0.75 *μ*L of 0.1 M MgCl_2_, and 32 *μ*L of ddH_2_O. Thermal cycling profile was conducted as follows: a first step at 95°C for 4 min, then 30 cycles of 95°C for 1 min, 58°C for 0.5 min and 72°C for 2 min, and a final step at 72°C for 8 min. Then the products were sent for sequencing to Quintarabio, Wuhan, China. The 16S rDNA sequence of strain LMM15 was compared in the NCBI GenBank using the BLAST; then the sequences of strains with high similarities were used for the construction of the phylogenetic tree by the Maximum Likelihood method using Molecular Evolutionary Genetics Analysis (MEGA) software version 6.0 [44].

### 2.8. Statistical Analysis

Calculation and comparison of treatment means for experiments were analyzed by the analysis of variance (ANOVA) using SPSS 22.0. All experiments described in this study involved three replicates per treatment. And all treatments were randomly placed. The data were subjected to means separation by Duncan's multiple range test, and statistical significance was applied at the level *p* = 0.05.

## 3. Results

### 3.1. Identification by 16S rDNA Gene Sequence Analysis

**Figure 1:**
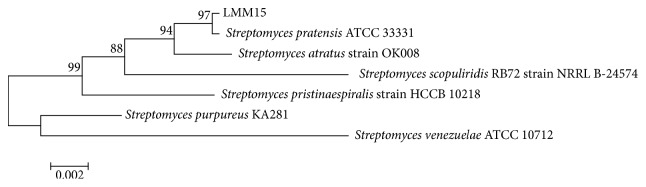
Phylogenetic relationship between the strain LMM15 and representative species based on full-length 16S rDNA sequences constructed using the neighbor-joining method. The number at each branch is the percentage of times the group of strains in that branch occurred, based on 1000 cycles in bootstrap analysis.

A neighbor-joining dendrogram was generated using the sequence from the strain LMM15 (1364 bp) and representative sequences from the databases. Phylogenetic analysis of 16S rDNA sequences of the strain LMM15 matched with the genus* Streptomyces*. The strain LMM15 had a maximum sequence similarity (97%) with* Streptomyces pratensis* (Figure 1).

### 3.2. Effects on Mycelial Growth of* B. cinerea*

**Figure 2:**
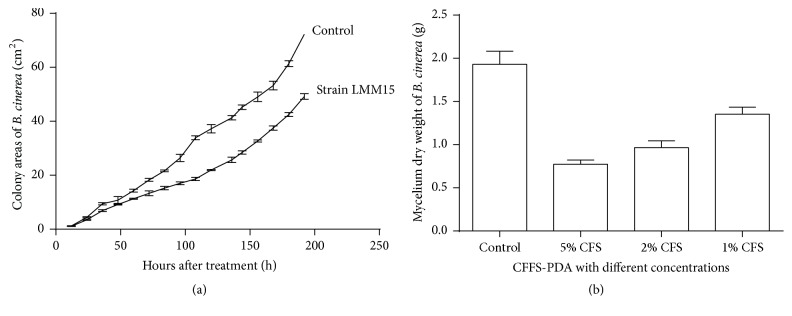
The antifungal effect of LMM15 to* B. cinerea.* (a) The surface areas of* B. cinerea* across different times spent on the containing plate. (b) The mycelium dry weight (DW) of* B. cinerea* in different CFFS-PDB.

The results in vitro of the 10%-CFFS-PDA and 5%, 2%, and 1%-CFFS-PDB experiments indicated that the CFFS of strain LMM15 strongly inhibited the hyphal growth of* B. cinerea* (Figure 2(a)). In the experiment of 10%-CFFS-PDA, the inhibitory rate of CFFS on* B. cinerea* gradually increased with the time of inoculation. When the hypha of the control covered the whole plate, the relative area of the* B. cinerea* colony was only 30.4% that of the control.

As to the CFFS-PDB experiment (Figure 2(b)), the mycelium dry weight (DW) of all treatments have been shown to significantly decline due to the inhibitory effect of the CFFS-PDB with different concentrations, as shown by the lowest value in the 5%-CFFS-PDB, the rate of decline being as high as 60.0%; then the rate of decline in 2%-CFFS-PDB was subsequently 50.1%.

### 3.3. The Biocontrol Efficacy In Vivo

**Figure 3:**
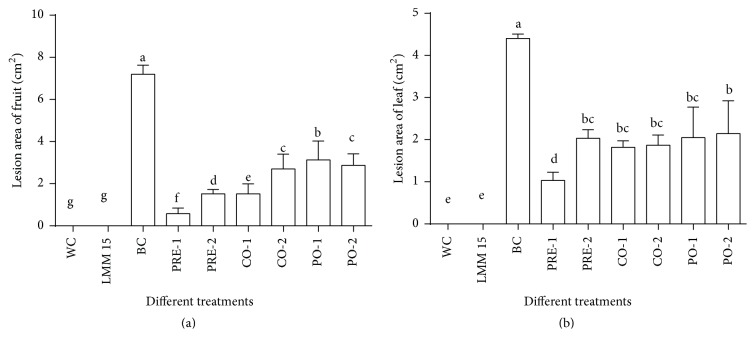
The biocontrol effect of LMM15 in vivo. WC: water control; BC:* Botrytis cinerea* only; PRE-1: pretreatment with LMM15, LMM15 was treated 24 hours before* B. cinerea*; PRE-2: pretreatment with procymidone, procymidone was treated 24 hours before* B. cinerea*; CO-1: cotreatment with LMM15, LMM15 was treated at the same time with* B. cinerea*; CO-2: cotreatment with procymidone, procymidone was treated at the same time with* B. cinerea*; PO-1: posttreatment with LMM15, LMM15 was treated 24 hours after* B. cinerea*; PRE-2: posttreatment with procymidone, procymidone was treated 24 hours after* B. cinerea*. Different lowercase letters on the same columnar indicate a significant difference at the 0.05 level. (a) is the result of fruits. (b) is the results of leaves.

The results of different treatments in vitro showed that strain LMM15 reduced decay incidence of gray mold on tomato fruits stored at 22°C for 3 days after inoculation (Figure 3(a)). The lesion area of gray mold was the lowest when the samples were treated with LMM15 24 hours before fungal inoculation (pretreatment), the efficacy was as high as 91.89%, and there was no significant difference with the effect of procymidone. The efficacy of LMM15 (57.91%) was higher than procymidone (52.78%) when the samples were treated with LMM15 or procymidone 24 hours after fungal (postpone-treatment) (Table 1). 

The gray mold assays proved that the LMM15 inoculation in different time conduct distinguished in extension of* B. cinerea* on the leaves in vitro (Figure 3(b)). Among the three timings of inoculated treatments, the application of LMM15 24 hours before fungus (pretreatment) had a better control, and the efficacy (76.64%) was lower than chemical agents (83.18%). When the samples were treated with the two agents at the same time with the fungus (cotreatment), the efficacy of LMM15 (58.62%) was higher than the procymidone (57.50%). There was no significant difference in control efficacy between LMM15 and the chemical agent when the samples were treated with LMM15 or procymidone 24 hours after fungal inoculation (postpone-treatment) (Table 1). 

### 3.4. Effects on the Plants Growth Promoting

The results showed that the fermentation broth of LMM15 treatment showed significantly higher levels in the case of the physiological indices of tomato plants (Table 2). The plant height was 12.19 centimeter 45 days after treatment with LMM15. While it was only 9.08 centimeters in the control, the increased amplitude was as high as 34.3%. When it comes to the ratio of DW/FW, it was only 0.137, while it was 0.141 in the treatment. The content of chlorophyll is related to the intensity of photosynthesis directly; when treated with LMM15, the content of chlorophyll was 25.0% higher than in the control. All of the above data reflect that LMM15 had significant effect on plants growth promoting, and this has already been described with other BCAs [45].

### 3.5. The Disease Control Efficacy

**Figure 4:**
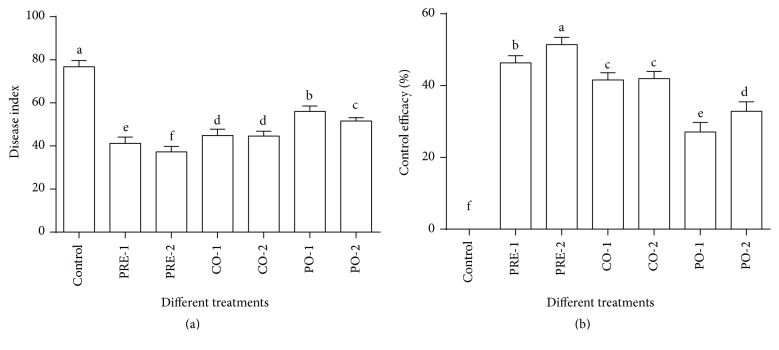
Control effects of LMM15 on tomato gray mold.

The disease control values of tomato plants treated with LMM15 or the chemical fungicide (procymidone) were compared after inoculation for 30 days (Figure 4). LMM15 and procymidone treatments were simultaneously carried out before and after inoculation of* B. cinerea* on tomato plants, which revealed the preventive and therapeutic efficacy of LMM15 and procymidone against tomato gray mold. The LMM15 treatment 24 hours before* B. cinerea* inoculation (pretreatment, HBI) produced a 46.35% disease control value, slightly lower than the chemical fungicide, whereas the LMM15 treatment inoculated with the pathogen (cotreatment) simultaneously showed the same control efficacy with the chemical fungicide (41.6% and 41.9%, resp.). However, the efficacy of treatment with LMM15 (only 27.0%) 24 hours after pathogen introduction (postpone-treatment) was much lower than that of the chemical fungicide.

### 3.6. Effect on the Plants Growth Promoting under* B. cinerea* Stress

The changes in the physiological factors of tomato plants indicated that the application of strain LMM15 significantly promoted plant growth under the stress of gray mold, including specific performance of its role of improvement in plant height, stem diameter, the ratio of DW/FW, and the total chlorophyll content (Table 3). From the casual morphological observations, the effects of strain LMM15 promoting plant height and stem diameter were higher than those of procymidone when plants samples were treated with LMM15 or procymidone 24 hours before pathogenic fungal inoculation. The improvement on the ratio of DW/FW was more outstanding than that of procymidone in all treatments with three different timings of inoculation.

The proline content in leaves reflects the resistance of the plants. The results (Table 4) show that the proline content of tomato leaf tissue underwent varying degrees of increase, especially with the pretreatment by LMM15 when the plants were under the stress of gray mold, and the proline content of the LMM15 treatments given before or simultaneously with inoculation of* B. cinerea* was higher than that of the procymidone treatment.

In addition, the MDA content reflects the stress severity of plants. The data expressed that the MDA contents have shown a significant decline compared to the pathogenic fungal control. The contents of LMM15 treatments before (22.86 *μ*mol/g·Fw) or simultaneously (26.81 *μ*mol/g·Fw) with inoculation were lower than that for the water control and even for the procymidone control in the same period.

### 3.7. The Activities of Defense-Related Enzymes

**Figure 5:**
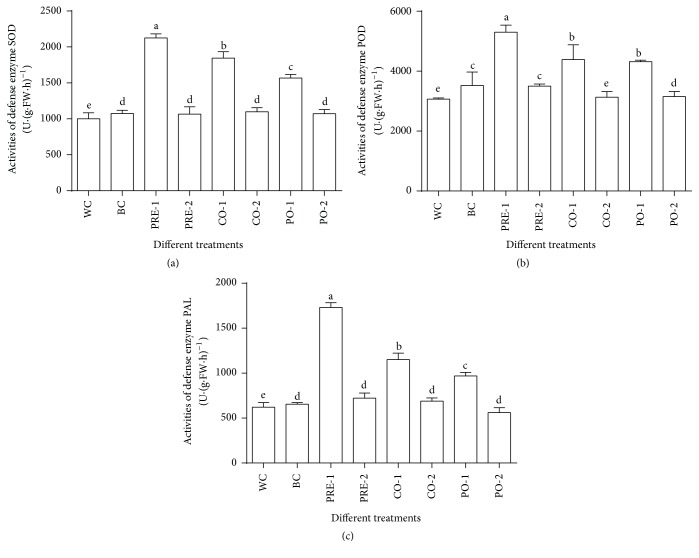
The influence on activity of defense enzymes of LMM15 on tomato plant.

To evaluate the changes in defense-related enzymes in tomato leaves, different enzymes levels, SOD, POD, and PAL activities in response to LMM15 are determined (Figure 5). Pretreatment with strain LMM15 dramatically induced SOD activity, being 2.2, and 2.1-fold higher than that of the water control or pathogenic fungal control. As for POD, the change in activity in response to the strain LMM15 showed a pattern similar to SOD. The POD activity in pretreatment with LMM15 was 1.5, and 1.2 times higher than for the water or pathogenic fungal control, and it was 3.1 and 2.9 times that for PAL activity.

## 4. Discussion

The phylogenetic analysis based on 16S rDNA showed that the strain LMM15 was 100% query cover and 97% identity with* Streptomyces pratensis* ATCC 33331 (Figure 1). The* S. pratensis* strain ATCC 33331 was isolated from grassy fields, it was formerly classified as* Streptomyces flavogriseus,* and then it was reclassified to* Streptomyces pratensis* sp. nov. based on 16S rDNA, atpD, gyrB, recA, rpoB, and trpB sequence [46]. In this study LMM15 was isolated from the tomato leaf which was infected by tomato leaf mold. This is the first report that LMM15, a* Streptomyces pratensis* strain, worked as biological agent to control tomato gray mold.

Tomato gray mold, caused by* B. cinerea,* is a devastating disease in tomato. Nowadays, many studies have shown that some beneficial microorganisms, as BCAs, are able to inhibit tomato gray mold, like* Streptomyces*,* Bacillus,* and some fungi [19–28]. In this study, the strain LMM15 as a BCA to control tomato gray mold was researched. The efficacy on mycelial growth showed that the strain LMM15 could significantly inhibit* B. cinerea* in vitro. The biocontrol efficacy on leaves and fruits and the tomato seedlings as well as growth promotion showed the strain LMM15 could significantly inhibit tomato gray mold (Figure 3) and strongly promote tomato plants growth. The gray mold disease control efficacy of preinoculation of strain LMM15 on potted seedlings was 46.35%.

The plants' normal physiology would be influenced when plants were under adversity stress [47], the balance among the production of reactive oxygen intermediates (ROI), and the activities of SOD, POD, or PAL and even antioxidants were disturbed [48]. Generally, injury, pathogen invasion, or environmental stress increase the activities of plants' defense-related enzymes, and the increased activities of SOD, POD, and PAL may strengthen the defense systems of plants [38]. Application of BCA could also increase the POD and PAL activities in plant organs [49, 50]. In our study, before treatment with LMM15 dramatically induced SOD activity, it was 2.2- and 2.1-fold higher than that of the water control or pathogenic fungal control. As to POD, the change in activity in response to the strain LMM15 showed a pattern similar to SOD. The PAL activity in pretreatment with LMM15 was 3.1 and 2.9 times higher than the PAL activity of tomato seedlings in the water control or pathogenic fungal control, respectively.

When the plants were under adversity stresses, the excessive ROI accumulated and resulted in the peroxidation occurring easily in intracellular membranes of plants. The sustained accumulation of ROI may cause the peroxidation damage and increase the malondialdehyde (MDA) content [51–53]. So the MDA content indicated the level of stress-induced damage to cell membranes [54]. Li et al. reported that the application of biocontrol agents could reduce the MDA content in the tissues compared to control [40]. In this study, when the tomato plants were treated by gray mold, MDA content increased quickly and reached to 85.19 *μ*mol/g·Fw. When the tomato plants were pretreated by LMM15 and then inoculated by* B. cinerea*, the MDA content was only 22.86 *μ*mol/g·Fw, which was less than any other treatments.

When under stress conditions, the proline in plant tissues may increase and protect the stability of enzymes as well as prevent membrane damage and protect the normal functioning of the ribosomes [55, 56]. In this study, the proline content of the tomato seedlings increased significantly when the plants were pretreated by LMM15 before the inoculation of gray mold. So LMM15 could protect tomato from* B. cinerea*.

Normally defense-related enzymes activities and proline content were much higher and the disease severity of gray mold and lipid membrane peroxidation levels were lower in robust plants. It is clear that there is a relationship among plant physiological response, incidence of disease, and defense-related enzyme systems. Our result showed that the activities of POD, SOD, and PAL were increased in tomato seedlings after treatment with LMM15; proline content was increased and MDA content was declined after treatment with LMM15, which all meant that LMM15 could induce the resistance to gray mold on tomato seedlings. So* S. pratensis* LMM15 could be a potential agent for controlling tomato gray mold.

## Figures and Tables

**Table 1:** The biocontrol efficacy in vivo.

	Leaf	Fruit
Pretreatment LMM15	91.89 ± 2.57^a^	76.64 ± 2.82^b^
Pretreatment procymidone	92.78 ± 1.38^a^	83.18 ± 2.73^a^
Cotreatment LMM15	58.86 ± 1.82^c^	58.62 ± 1.93^c^
Cotreatment procymidone	61.24 ± 2.86^b^	57.50 ± 2.14^c^
Postpone-treatment LMM15	57.91 ± 1.47^c^	49.32 ± 1.69^d^
Postpone-treatment procymidone	52.78 ± 2.62^d^	50.26 ± 2.41^d^

*Note*. The data are the means of three independent experiments, each with three replications. Different lowercase letters in the same column indicate a significant difference at the 0.05 level.

**Table 2:** The growth promoting effect on tomato seedlings by fermentation liquid of LMM15.

	Plant height (cm)	Stem diameter (cm)	The ratio of DW/FW	The total chlorophyll content (mg/g·Fw)
Water control	9.08 ± 0.102	0.635 ± 0.001	0.137 ± 0.000	1.27 ± 0.023
LMM15	12.19 ± 0.326	0.656 ± 0.001	0.141 ± 0.001	1.59 ± 0.085

*Note*. The data are the means of three independent experiments, each with three replications.

**Table 3:** The growth promoting effect on tomato seedlings by fermentation liquid of LMM15 under the stress of *Botrytis cinerea*.

	Water control	*B. cinerea *only	Pretreatment		Cotreatment		Postpone-treatment	
			LMM15	Procymidone	LMM15	Procymidone	LMM15	Procymidone
Plant height (cm)	18.17 ± 0.22^c^	16.67 ± 0.26^e^	20.57 ± 0.19^a^	19.00 ± 0.15^b^	18.30 ± 0.21^c^	18.53 ± 0.20^bc^	17.20 ± 0.17^de^	17.37 ± 0.23^d^
Stem diameter (cm)	0.527 ± 0.008^a^	0.499 ± 0.008^c^	0.513 ± 0.009^a^	0.542 ± 0.008^a^	0.512 ± 0.006^b^	0.514 ± 0.008^b^	0.501 ± 0.010^c^	0.500 ± 0.008^c^
The ratio of DW/FW	0.135 ± 0.001^bc^	0.127 ± 0.000^e^	0.140 ± 0.001^a^	0.141 ± 0.001^a^	0.136 ± 0.000^b^	0.133 ± 0.001^cd^	0.131 ± 0.000^d^	0.130 ± 0.000^de^
Chlorophyll content (mg/g·Fw)	1.909 ± 0.010^ab^	1.633 ± 0.010^g^	1.928 ± 0.008^a^	1.885 ± 0.020^b^	1.814 ± 0.010^d^	1.843 ± 0.020^c^	1.721 ± 0.008^f^	1.779 ± 0.008^e^

*Note*. The data are the means of three independent experiments, each with three replications. Different lowercase letters in the same column indicate a significant difference at the 0.05 level.

**Table 4:** The effect on the content of Pro and MDA by fermentation liquid of LMM15 under the stress of *Botrytis cinerea*.

	Proline content (ug/g)	MDA content (umol/g·Fw)
Water control	47.09 ± 2.57^f^	50.28 ± 1.00^c^
*B. cinerea *only	51.70 ± 4.34^ef^	85.19 ± 1.65^a^
Pretreatment LMM15	127.20 ± 1.57^a^	22.86 ± 1.82^f^
Pretreatment procymidone	110.28 ± 2.88^b^	26.81 ± 1.73^e^
Cotreatment LMM15	94.97 ± 3.22^c^	30.72 ± 1.59^d^
Cotreatment procymidone	62.37 ± 3.71^d^	32.61 ± 1.41^d^
Postpone-treatment LMM15	54.49 ± 0.37^e^	72.52 ± 1.99^b^
Postpone-treatment procymidone	60.49 ± 2.02^d^	52.26 ± 1.78^c^

*Note*. The data are the means of three independent experiments, each with three replications. Different lowercase letters in the same column indicate a significant difference at 0.05 level.
